# Lipopolysaccharide promotes lipid accumulation in human adventitial fibroblasts via TLR4-NF-κB pathway

**DOI:** 10.1186/1476-511X-11-139

**Published:** 2012-10-17

**Authors:** Jun Wang, Yanfang Si, Chen Wu, Lu Sun, Yudong Ma, Aili Ge, Baomin Li

**Affiliations:** 1 Department of Neurosurgery, the General Hospital of PLA, Beijing, 100853, China; 2 Department of Ophthalmology, the 309th Hospital of Chinese PLA, Beijing, 100091, China; 3 Department of Pathology, the General Hospital of PLA, Beijing, 100853, China

**Keywords:** LPS, Lipid accumulation, ADRP, Atherosclerosis, Adventitial fibroblasts, Toll-like receptor 4

## Abstract

**Background:**

Atherosclerosis is a chronic degenerative disease of the arteries and is thought to be one of the most common causes of death globally. In recent years, the functions of adventitial fibroblasts in the development of atherosclerosis and tissue repair have gained increased interests. LPS can increase the morbidity and mortality of atherosclerosis-associated cardiovascular disease. Although LPS increases neointimal via TLR4 activation has been reported, how LPS augments atherogenesis through acting on adventitial fibroblasts is still unknown. Here we explored lipid deposition within adventitial fibroblasts mediated by lipopolysaccharide (LPS) to imitate inflammatory conditions.

**Results:**

In our study, LPS enhanced lipid deposition by the up-regulated expression of adipose differentiation-related protein (ADRP) as the silencing of ADRP abrogated lipid deposition in LPS-activated adventitial fibroblasts. In addition, pre-treatment with anti-Toll-like receptor 4 (TLR4) antibody diminished the LPS-induced lipid deposition and ADRP expression. Moreover, LPS induced translocation of nuclear factor-κB (NF-κB), which could markedly up-regulate lipid deposition as pre-treatment with the NF-κB inhibitor, PDTC, significantly reduced lipid droplets. In addition, the lowering lipid accumulation was accompanied with the decreased ADRP expression. Furthermore, LPS-induced adventitial fibroblasts secreted more monocyte chemoattractant protein (MCP-1), compared with transforming growth factor-β1 (TGF-β1).

**Conclusions:**

Taken together, these results suggest that LPS promotes lipid accumulation via the up-regulation of ADRP expression through TLR4 activated downstream of NF-κB in adventitial fibroblasts. Increased levels of MCP-1 released from LPS-activated adventitial fibroblasts and lipid accumulation may accelerate monocytes recruitment and lipid-laden macrophage foam cells formation. Here, our study provides a new explanation as to how bacterial infection contributes to the pathological process of atherosclerosis.

## Background

Atherosclerosis Â is considered to be one of the most common causes ofÂ death globally due to higher morbidity and mortality Â in brain and heart, such as cerebral infarction. Atherosclerosis is a chronic degenerative disease of theÂ arteries, representing the root cause of the majority ofÂ cardiovascular disease (CVD) and their complications,Â including coronary artery disease and myocardial infarction [1-3]. It is a complex inflammatory process that is characterized by the accumulation of lipids and fibrous elements in arteries [4].

In recent years, a relationship between adventitia and atherosclerosis has garnered increasing interests [5,6]. Fibroblasts, as a major component of adventitia, are thought to be the critical contributor to adventitial function in vascular inflammation, remodeling and neovascularization. The action of this roles depend on the secretion of many proinflammatory cytokines such as interleukin (IL-6), reactive oxygen species (ROS) and MCP-1, which are strongly correlated with advanced atherosclerosis [7,8]. It has been demonstrated that recurrent injuries and repairs elicit the proliferation and rearrangement of fibroblasts, finally leading to tissue alteration in many chronic inflammatory diseases [9,10]. Although the importance of adventitial fibroblasts in atherosclerosis has gained widespread acceptance, little is known about the precise signaling pathways.

Lipopolysaccharide (LPS), a component of Gram-negative bacteria cell walls, is thought to be involved in cardiovascular disease as contribution to the development of arterial plaques through activated proinflammatory pathways [11-14]. Acting on adventitial fibroblasts, LPS can cause intima hyperplasia via the activation of the NF-κB pathway to secrete cytokines, including IL-6, MCP-1 and tumor necrosis factor-α (TNF-α) [14]. Additionally, injection of bacterial endotoxin-LPS in apolipoprotein E (apoE)-deficient mice increases atherosclerotic lesion size [11]. Toll-like receptors (TLRs) are key components of the innate immune system in atherosclerosis-based pathology. Deficiency in TLR4, the receptor of LPS, reduces aortic atherosclerosis in apoE-deficient mice [15]. Reduced atherosclerosis can also be observed by injecting with melittin, which abolishes the expression of LPS-induced TNF-α and IL-1β and inhibits NF-κB signal [16].

Lipid deposition is thought to be a major risk factor for diseases ranging from obesity to atherosclerosis [7]. An excess of lipid-laden macrophage foam cells formation results from lipid overproduction and is a hallmark of atherosclerosis, forming the earliest detected lesion, the fatty streak [4]. Aggressive lowering of lipid levels markedly reduces atherosclerotic coronary lesion and has thus attracted more attention as potential therapeutic targets [17-19]. Adipose differentiation-related protein (ADRP), a major lipid droplet protein, regulates foam cell formation and atherosclerotic development. Its absence severely restricts foam macrophage cell formation and attenuates atherosclerosis [18,20].

To explore the role of adventitial fibroblasts in the pro-atherosclerotic effects as lipid accumulation during infection, we investigated whether LPS stimulation regulated lipid accumulation via ADRP expression in adventitial fibroblasts, the related signal pathways and induced MCP-1 were also analyzed in this process.

## Methods

### Preparation of ox-LDL

Human LDL was obtained as described previously [21]. For the production of ox-LDL, 200 μg/ml LDL was exposed to 20 μM CuSO_4_ in phosphate buffered saline (PBS) for oxidation and the reaction was stopped with 40 μM butylhydroxytoluene in ethanol. The oxidized LDL (CuoxLDL) was then dialyzed against culture medium and sterilely filtered.

### Cell culture and treatments

Primary adventitial fibroblasts were isolated from human aortas. All human material was obtained and processed according to the recommendation of the Fourth Military Medical University, China. The study was conducted in compliance to the Helsinki Declaration, and all patients gave written informed consent for publication of this report. Isolated cells were cultured in stromal cell growth medium (SCGM) containing 5% fetal bovine serum (FBS), and maintained at 37°C and 5% CO_2_. Cells of passages 4 to 9 were used. These cells were obtained from normal aortic tissue and their identity and purity were confirmed based on morphology and growth characteristics as previously described [22]. All tissue culture components and solutions were purchased from Gibco BRL (Paisley, UK).

Cells were treated with different stimuli and divided into two groups. One group was treated with, or without, LPS (10 μg/ml) (Sigma, St. Louis, MO) [21,23] or TGF-β1 (10 μg/ml) (GenWay Biotech, San Diego, CA). The other group was separately pre-treated with anti-TLR4 antibody (10 μg/ml) (eBioscience, San Diego, CA) for 2 h, 25 μM PDTC (NF-κB inhibitor, Calbiochem, La Jolla, CA) for 2 h, and then exposed to 10 μg/ml LPS. The normal adventitial fibroblasts were used as control group cultured without any treatment. All cells were incubated in the CO_2_ incubator before being used in assays. After culturing for different time periods, cells were harvested.

### Lipid analysis by high-performance liquid chromatography (HPLC)

Cellular total cholesterol (TC) and cholesterol ester (CE) contents in treated and control cells were analyzed by HPLC. Briefly, after incubation with CuoxLDL, cells were washed 3 times by PBS, lysed by 0.9% NaOH solution and homogenized on ice for 10 seconds by ultrasound. Protein concentration was measured by the BCA kit (Pierce, Rockford, IL). Then, an equal volume of trichloroacetic acid was added and centrifuged at 800×g for 10 min. Masterol was used as a standard curve first, and the extraction procedure was then repeated. The samples were dissolved in 100 μl of isopropanol–acetonitrile (v/v, 20:80), followed by incubation in an ultrasound water bath at room temperature for 5 minutes. Finally, the samples were placed in the Agilent 1100 series HPLC (Agilent Technology, Palo Alto, CA).

### Quantitative real-time PCR

Total cellular RNA was extracted from human adventitial fibroblasts using TRIZOL reagent (Sigma). Total RNA (~ 4 μg) was reverse transcribed to synthesize first strand cDNA with the Oligo (dT) _18_ primer using the cDNA Synthesis Kit (Fermentas, St. Leon-Rot, Germany). Then, 2 μl of cDNA was used as a template in the PCR reaction mixture with gene-specific primers (forward 5^′^-TGCACTCACCAAATCAGAGC-3^′^ and reverse 5^′^-AAGGGACCTACCAGCCAGTT-3^′^ for ADRP (GenBank accession no. BC005127.2)). For normalization, β-actin mRNA was used. SYBR Green I was used as the fluorochrome in real-time PCR amplification. The reaction conditions followed the instructions provided by the manufacturers of the SYBR Premix Ex Taq^TM^ II Kit (Takara Bio Inc., Otsu, Japan).

### Western blotting

Immunoblotting analysis of ADRP was performed on fibroblasts treated or not with LPS for 24 h. Briefly, harvested cells were washed twice with ice-cold PBS, lysed with NE-PERTM or MEM-PERTM protein extraction reagent (Pierce), and supplemented with a protease inhibitor cocktail (Sigma). The collected protein concentrations were determined by the BCA assay (Pierce) and their proteins were electrophoresed on a 12% polyacrylamide gel and transferred onto a polyvinylidene difluoride (PVDF) membrane. After blocking, the target proteins were probed with anti-NF-κB p65 (1:1000) or anti-ADRP (1:1000; Fitzgerald Industries Intl, Concord, MA) overnight at 4°C, and then HRP-conjugated anti-mouse antibodies (Sigma, St. Louis, Mo.) were added at room temperature for 1 h. The bound antibodies were visualized by using the LumiGLo reagent (Pierce) and the levels of each protein relative to that of the β-actin were analyzed.

### Silencing of ADRP expression with small interference RNA (siRNA)

To design target-specific siRNA, specific fragments of ADRP and the Scramble II control were designed using the internet tool of siRNA wizard (Invivogen, San Diego, CA). The cDNA sequence of the Scramble II control was 5’-AAGCGCGCUUUGUAGGAUUCG-3’ (21 nt). The sense and antisense strands with two base overhangs were synthesized (GeneChem, Shanghai, China). Human adventitial fibroblasts (6×10^5^/ml) were transfected with 2 μg/ml ADRP siRNA or Scramble II siRNA using the GeneSilencer® siRNA transfection reagent (GeneTherapy System, San Diego, CA), according to the manufacturer’s instructions. About 24 h later, cells were washed and then incubated with LPS for 24 h.

### ELISA analysis

To evaluate the produced levels of MCP-1, either TGF-β1 or LPS was added to the medium to induce fibroblast differentiation. In short, cells were seeded at a density of 50×10^3^ cells per well and stimulated for 24 h. The supernatant was harvested and the expression levels of MCP-1 were detected by the Human MCP-1 ELISA Kit (R&D Systems, Minneapolis, MN) according to the manufacturer’s instructions.

### Data analysis

Data were analyzed using SPSS 11.0 software. A typical image from at least three similar experiments was presented. Statistical analysis was carried out using *t*-tests. P < 0.05 was considered statistically significant. All results are expressed as mean ±SD.

## Results

### LPS-stimulation enhanced lipid accumulation

**Figure 1:**
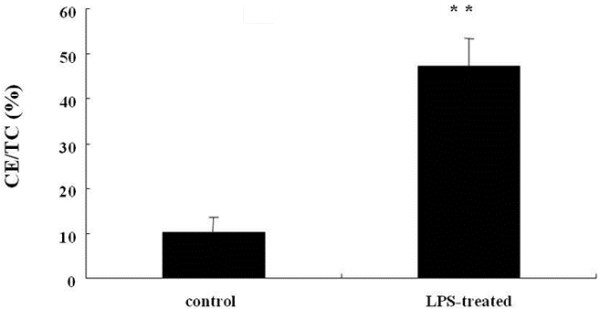
**LPS induced lipid deposition in the activated adventitial fibroblasts.** CuoxLDL was added to LPS-induced, or not, cells. The ratio of CE/TC was used to evaluate lipid accumulation. ** *P* < 0.01.

LPS is a major contributor to the development of arterial plaques through activated proinflammatory pathways [11-14]. To ascertain whether LPS can induce lipid deposition in adventitial fibroblasts, CuoxLDL was added and the ratio of CE/TC was used to analyze lipid accumulation. As shown in Figure 1, the values of CE/TC were 10.1±3.5% in the CuoxLDL control group and 47.2±6.2% in the LPS-CuoxLDL group. This showed that LPS-stimulated adventitial fibroblasts accelerated the uptake of CuoxLDL and promoted cholesterol ester deposition, compared to the control group. 

### LPS induced the lipid deposition via up-regulating the expression of ADRP in adventitial fibroblasts

**Figure 2:**
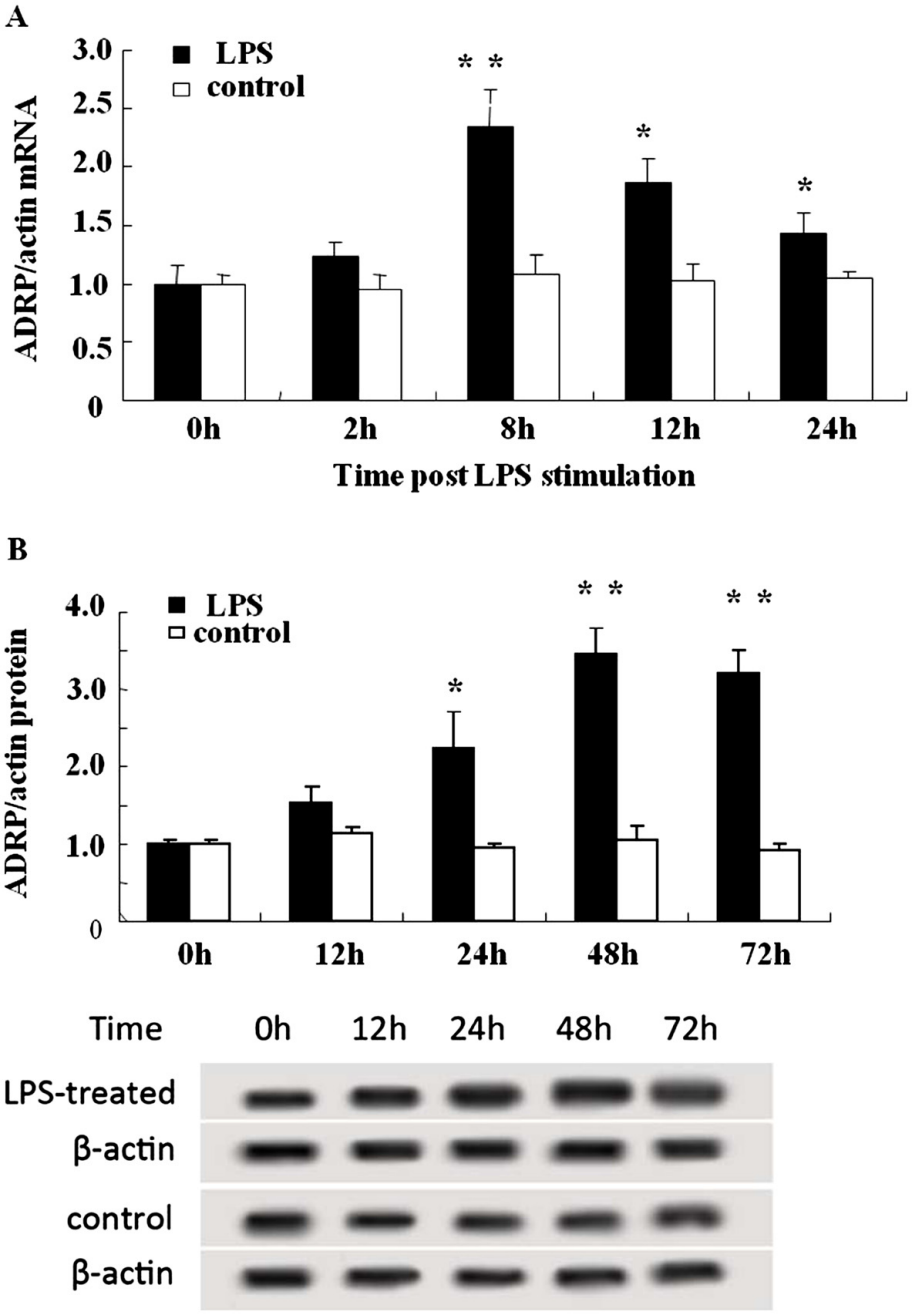
**LPS up-regulated the expression levels of ADRP mRNA and protein.** After stimulation with, or without, LPS (10 μg/ml) for 0 to 48 hours, ADRP mRNA and protein levels were analyzed. (**A**) LPS-induced expression of ADRP mRNA. (**B**) The corresponding protein levels of ADRP in LPS-induced fibroblasts. ** *P* < 0.01. *P < 0.05.

As a major lipid droplet protein, ADRP plays crucial roles in regulating foam cell formation and atherosclerotic development, and is abundant in lipid-laden cells [18,20]. Therefore, to understand that how LPS promotes lipid accumulation, ADRP was analyzed here. After stimulation with LPS for different time periods, ADRP mRNA and cellular protein levels were analyzed by real-time PCR and Western blotting, respectively. Compared to the control group, a significant up-regulation of ADRP mRNA was confirmed at 8 h after LPS stimulation, which then gradually decreased (Figure 2A). Consistent with the above observation, LPS also induced a rapid increase in ADRP protein level (Figure 2B), but this lagged behind the expression of ADRP mRNA. The expression of ADRP protein was notably induced by LPS stimulation and was about 3.5-fold higher than that of the untreated group at 48 h. All mRNA and protein level analyses showed that LPS significantly enhanced the expression of ADRP mRNA and protein. 

**Figure 3:**
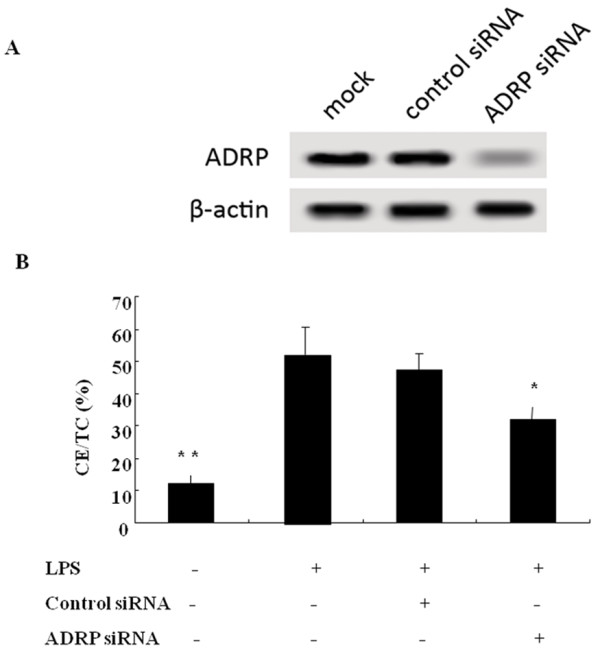
**Silencing of ADRP decreased lipid deposition in LPS-activated fibroblasts.** Cultured cells were transfected with 2 μg/ml of ADRP siRNA or Scramble II siRNA before exposure to LPS. The effect of silencing ADRP was analyzed by Western blotting (**A**) and the resulting lipid accumulation was characterized by HPLC (**B**).

Whether ADRP is the contributor to lipid accumulation during LPS stimulation, to address this question, the expression of ADRP was silenced by siRNA targeting ADRP and western blotting was used to evaluate the silencing effect of ADRP in LPS-activated cells. As shown in Figure 3A, most of the expression of ADRP was silenced; the absence of ADRP strikingly reduced lipid accumulation and the ratio of CE/TC. However, the ratio of CE/TC in the ADRP siRNA pre-treated cells was still higher than that of the LPS-untreated group (Figure 3B). All of these results suggested that LPS could promote lipid deposition via the up-regulating ADRP expression, but it was not the only molecule involved in this process.

### LPS-induced lipid deposition depended on the activation of TLR4 and NF-κB pathway

**Figure 4:**
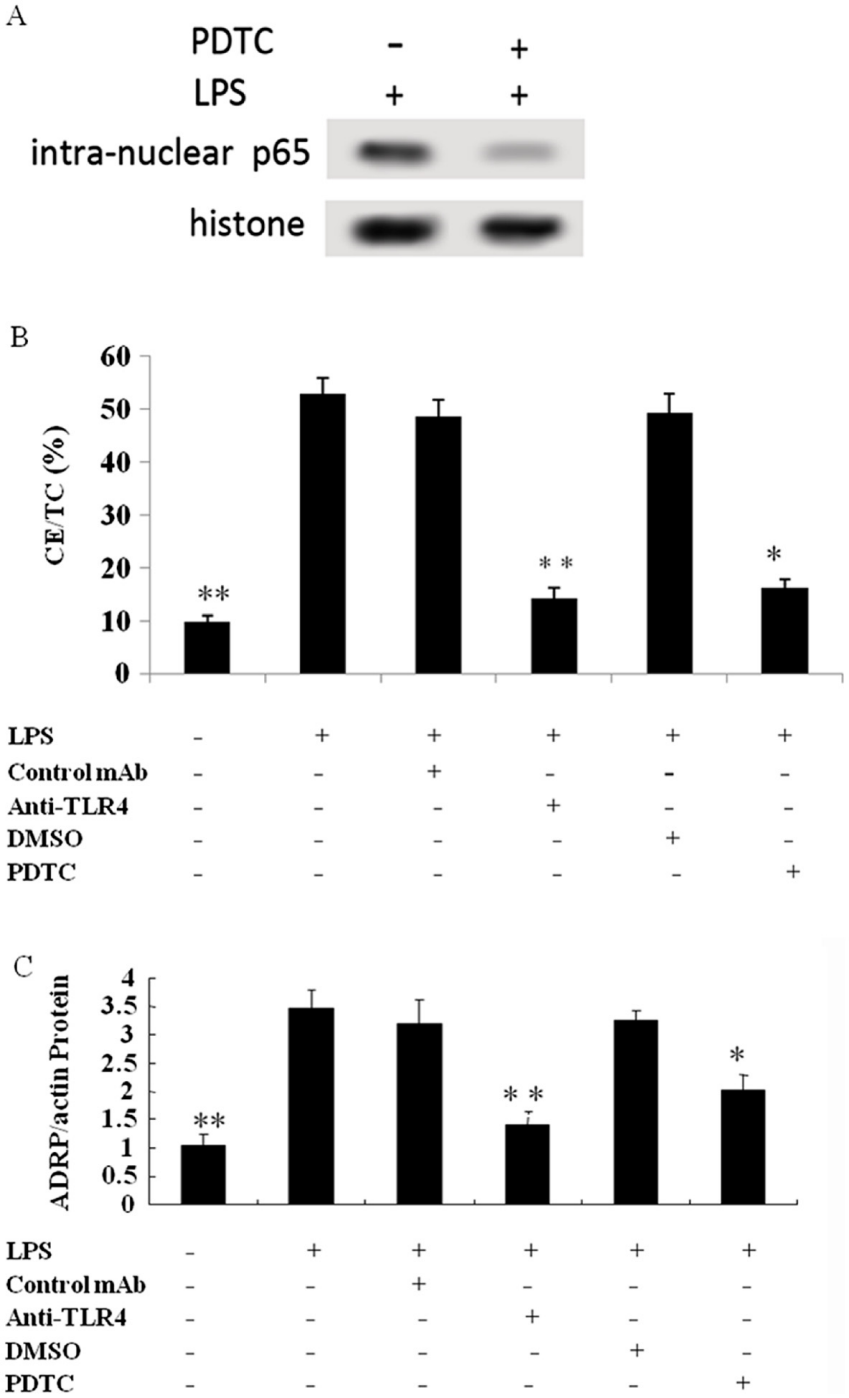
**The induced lipid accumulation resulted from up-regulated expression of ADRP via LPS-activated TLR4 and NF-κB pathway.** Cultured adventitial fibroblasts were pretreated with or without anti-TLR 4 antibodies and the NF-κB inhibitor PDTC for 1 h before exposure to 10 μg/ml of LPS for 48 h. Compared to the control group, treatment with the inhibitors significantly abrogated the LPS-induced increase in ADRP expression and lipid deposition. (**A**) The activation and inhibition of NF-κB pathway was analyzed by Western blotting. Cultured cells were pretreated with, or without, NF-κB inhibitor PDTC before LPS stimulation. (**B**) Induced lipid accumulation via the LPS-activated TLR4 and NF-κB pathway. (**C**) The induced lipid accumulation was accompanied with ADRP expression. The expression of ADRP mRNA in activated, or not, cells were analyzed by real-time PCR. ** *P* < 0.01. *P < 0.05.

As a receptor of LPS, TLR4 and its downstream signaling effectors, NF-κB, are pivotal in the initiation and development of atherosclerosis [15,24]. The intra-nuclear NF-κB p65 and control histone were characterized by Western blotting. The intra-nuclear translocation of NF-κB was obviously observed following LPS stimulation. At the same time, significant inhibition of NF-κB activation was confirmed by pretreatment with the NF-κB inhibitor, PDTC (Figure 4A). 

To analyze whether lipid accumulation was correlated with the activation of TLR4 and the downstream NF-κB pathway, real-time PCR was used here (Figure 4B). Obviously, pretreatment with the anti-TLR4 antibody significantly decreased lipid deposition in adventitial fibroblasts, suggesting that TLR4 was essential for LPS-induced lipid droplets. Furthermore, pretreatment with PDTC resulted in a corresponding attenuated lipid deposition in LPS-activated adventitial fibroblasts, compared with DMSO control (Figure 4B). As shown in Figure 4C, the lipid accumulation was accompanied with ADRP expression during LPS activated pathway. These results confirmed that the LPS could induce lipid deposition via ADRP expression through TLR4 and NF-κB signaling pathway in adventitial fibroblasts.

### Higher amounts of MCP-1 were induced by LPS than TGF-β1 treatment

**Figure 5:**
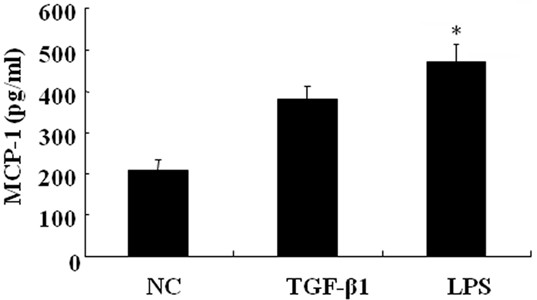
**Induced secretion of MCP-1 in activated adventitial fibroblasts pretreated with either LPS or TGF-β1.** The cells were incubated with, or without, the same concentration of stimuli for 24 h. The concentration of MCP in the cultured medium was determined by ELISA. Results are mean ±SD. *P < 0.05 compared to control cells (NC).

As described previously, activated adventitial fibroblasts can produce many cytokines and chemokines to affect the development of atherosclerosis [7,8]. Lots production of MCP-1 are induced by LPS and TGF-β1 stimulation in adventitial fibroblasts. To evaluate the activating ability of LPS and TGF-β1 on adventitial fibroblasts, the induced expression of MCP-1 was determined by ELISA analysis (Figure 5). Compared to the control group, LPS induced a statistically significant up-regulation of MCP-1 expression and a higher amount of MCP-1 was detected in the LPS-treated group compared to the TGF-β1 one. This showed that LPS could more easily induce the expression of MCP-1 than TGF-β1 in activated adventitial fibroblasts. 

## Discussion

Atherosclerosis is the major risk factor for human health, the related diseased has resulted in high mortality, including cerebral infarction and complications of cardiovascular disease [1]. Atherosclerosis is characterized with the accumulation of lipids and fibrous elements. Adventitial fibroblasts can transform into activated myofibroblasts, which have the ability to proliferate and migrate to the vascular lumen by the induced production of proinflammatory cytokines, chemokines and extracellular matrix (ECM). This switch of fibroblasts into myofibroblasts is important for vascular repair and atherosclerotic plaques formation [4,6,25]. Many factors can activate the transformation of adventitial fibroblasts into myofibroblasts. LPS, as one of these factors, has been discussed in many reports [26,27]. An epidemiological survey confirms that Gram-negative bacteria may increase the morbidity and mortality of atherosclerosis-associated cardiovascular disease [21,28,29]. Many factors can serve as a mediator/inducer of atherosclerosis by interaction with a common inflammation pathway-TLR4, including heat shock protein (HSP60) and LPS [11,30]. As a common ligand of TLR4, LPS is considered as crucial for the initiation and development of atherosclerosis [11-14]. However, LPS involvement in lipid accumulation in adventitial fibroblasts has not been reported. Here, we studied the molecular mechanism underlying LPS-mediated lipid accumulation and speculated the possible correlation between activated adventitial fibroblasts and the formation of foam cells.

Lipid deposition is a trigger for atherosclerosis and complications [7,18,19]. Foam cells are major components of atherosclerotic plaques and their formation mainly depends on lipid accumulation [31,32]. The cholesterol in foam cells is present mainly as cytoplasmic cholesteryl ester and marked accumulation of CE results in foam cell formation [33,34]. Genetic ablation of neutral CE hydrolase 1 (Nceh1) promotes foam cell formation and aggravates atherosclerosis in mice [35]. ADRP, as a PAT-domain protein, can promote lipid accumulation in macrophages and lipid-laden cells formation [18,20]. Oxidative modification of LDL is considered to promote arterial lipid accumulation and atherosclerosis [36]. In our study, LPS-activated adventitial fibroblasts could accelerate the ingestion of CuoxLDL and ultimately promote CE accumulation through a drastic increase in ADRP expression, but this was not the only molecule involved in LPS-mediated lipid deposition as the lipid droplet amount in ADRP-siRNA treated cells was still higher than that of LPS-untreated group.

As lots of CE production is more prone to form foam cells and is critical for atherosclerotic plaque induction. So it is very necessary for us to clarify that which signaling pathway was involved in LPS-mediated lipid accumulation. As the absence of the downstream adaptor molecule TLR4 is associated with reduced atherosclerotic plaque formation via the down-regulation of MCP-1and macrophages in the plaque [15]. Therefore, to address this question, TLR4 and its downstream signaling effectors, NF-κB, were investigated here. As a receptor of LPS, TLR4 is pivotal in the initiation and development of atherosclerosis [15,24]. An obvious correlation between lipid droplets and TLR4-NF-κB pathway was confirmed, because pre-treatment with anti-TLR4 antibody and PDTC inhibitor significantly abrogated the lipid deposition in LPS-activated adventitial fibroblasts. In addition, LPS-induced lipid accumulation is accompanied with ADRP expression during this process. Thus, it appears that LPS promotes lipid accumulation by increasing ADRP expression through TLR4 and activated downstream NF-κB pathway.

During atherosclerosis, the chemokine MCP-1 is an important component in the initiation of atherosclerotic plaques. It is widely accepted that MCP-1 can recruit monocytes by the surface receptor CCR2, accelerate cellular infiltration of lymphocytes and characteristically accumulate in nascent atheroma [37,38]. A mutant lacking MCP-1 or its receptor CCR2 shows striking decreases in mononuclear phagocyte accumulation and local lipid levels [39,40]. Many factors can activate the production of MCP-1, such as LPS, TGF-β1 and angiotensin. TGF-β1 is an important immunomodulatory cytokine which can significantly stimulate the expression of MCP-1 in fibroblast cells [41]. LPS can induce MCP-1 up-regulation by the activated NF-κB signaling pathway in fibrocyte [42]; the inhibition of this pathway results in a strong decrease *in vitro*[43]. However, there is no report about the comparative analysis of the two inducers regarding MCP-1 production. In this study, we found that LPS could strongly induce more secretion of MCP-1 in activated adventitial fibroblasts than TGF-β1, indicating that more monocytes could be recruited to regulate lipid accumulation by the transformation into macrophages.

## Conclusions

It is well known that lipid accumulation and activated macrophages are the basis of foam cells formation, which are triggers for atherosclerotic plaque production [7,17,31]. LPS can up-regulate the expression of Fcamr (Fc α/μ receptor) through activated NF-κB and the p38 MAPK pathway to increase the formation of lipid-laden foam cells [21]. Here, LPS stimulation can activate TLR4 in adventitial fibroblasts, thereby increasing ADRP expression through the NF-κB pathway to promote lipid accumulation. The absence of TLR4 can reduced atherosclerotic plaque formation via the down-regulation of MCP-1 [15]. MCP-1 can recruit monocytes and promote lipid-laden foam cells formation, lots of MCP-1 production was detected in LPS-stimulated cells in our study. Based on these results, we can conclude that LPS can induce lipid-laden foam cells formation via MCP-1 production and lipid deposition by TLR4-NF-κB pathway, and accelerate the pathogenesis of atherosclerosis. Taken together, our work may provide a new understanding about why bacterial infection increases the mortality of atherosclerosis-related cardiovascular diseases. In recent years, the NF-κB pathway has attracted increasing attention as a drug-development target for treating inflammatory diseases [44]. Thus, future studies should focus on how to slow down the development of atherosclerosis by targeting the bioactive molecules involved in the NF-κB pathway, such as ADRP.

## Abbreviations

ADRP: Adipose differentiation-related protein; LDL: Low-density lipoprotein; TLR4: Toll-like receptor 4; NF-κB: Nuclear factor-kappa B; TNF-α: Tumor necrosis factor-α; LPS: Lipopolysaccharide; MCP-1: Monocyte chemoattractant protein; TGF-β1: Transforming growth factor-β1; CVD: Cardiovascular disease; IL-6: Interleukin-6; ROS: Reactive oxygen species; SCGM: Stromal cell growth medium; FBS: Fetal bovine serum; ECM: Extracellular matrix; DMSO: Dimethyl sulfoxide; PDTC: Pyrrolidine dithiocarbamate; HSP60: Heat shock protein.

## Competing interests

The authors declare that they have no competing interests.

## Authors' contributions

All the authors were involved in the design of this study. JW and YFS substantially contributed to the design of the study, performing the experiment, analysis of data, and drafting the manuscript. CW and LS participated in ox-LDL preparation, cell culture and treatments. YM carried out lipid analysis and ADRP detection. AG carried out siRNA preparation, NF-κB,TLR-4 and MCP-1 assays. BML made contribution to design, analysis and revision of the manuscripts. All the authors have read and approved the final version.
